# Prehospital Misdiagnosed Acute Coronary Syndrome—Incidence, Discriminating Features, and Differential Diagnoses

**DOI:** 10.31083/j.rcm2403075

**Published:** 2023-03-02

**Authors:** Josefin Grabert, Ulrich Heister, Andreas Mayr, Tobias Fleckenstein, Andrea Kirfel, Christian Staerk, Maria Wittmann, Markus Velten

**Affiliations:** ^1^Department of Anaesthesiology and Intensive Care Medicine, University Hospital Bonn, 53127 Bonn, Germany; ^2^Emergency Medical Service Bonn, 53103 Bonn, Germany; ^3^Department of Medical Biometry, Informatics and Epidemiology, University Hospital Bonn, 53127 Bonn, Germany

**Keywords:** acute coronary syndrome, prehospital, misdiagnosis

## Abstract

**Background:**

Acute coronary syndrome (ACS) is a major cause of morbidity and mortality in the western world. Classic angina pectoris (AP) is a common reason to request prehospital emergency medical services (EMS). Nevertheless, data on diagnostic accuracy and common misdiagnoses are scarce. Therefore, the aim of this study is to evaluate the amount and variety of misdiagnoses and assess discriminating features.

**Methods:**

For this retrospective cohort study, all patients requiring EMS for suspected ACS in the city of Bonn (Germany) during 2018 were investigated. Prehospital and hospital medical records were reviewed regarding medical history, presenting signs and symptoms, as well as final diagnosis.

**Results:**

Out of 740 analyzed patients with prehospital suspected ACS, 283 (38.2%) were ultimately diagnosed with ACS (ACS group). Common diagnoses in the cohort with non-confirmed ACS (nACS group) consisted of unspecific pain syndromes, arrhythmias, hypertensive crises, and heart failure. ST segment elevation (adjusted odds-ratios [adj. OR] 2.70), male sex (adj. OR 1.71), T wave changes (adj. OR 1.27), angina pectoris (adj. OR 1.15) as well as syncope (adj. OR 0.63) were identified among others as informative predictors in a multivariable analysis using the lasso technique for data-driven variable selection.

**Conclusions:**

Misdiagnosed ACS is as common as 61.8% in this cohort and analyses point to a complex of conditions and symptoms (i.e., male sex, electrocardiographic (ECG) changes, AP) for correct ACS diagnosis while neurological symptoms were observed significantly more often in the nACS group (e.g., Glasgow Coma Scale (GCS) <15, *p* = 0.03). To ensure adequate and timely therapy for a potentially critical disease as ACS a profound prehospital examination and patient history is indispensable.

## 1. Introduction 

Ischemic heart disease and acute coronary syndrome (ACS) are major causes of morbidity and mortality in the western world, accounting for approximately 22% of global deaths [1]. Although the incidence of ACS shows regional differences, coherent clinical diagnosis is associated with chest pain, being one of the most common symptoms to demand emergency medical services (EMS), associated with up to 20% of all prehospital emergency operations [2]. However, most patients presenting with chest pain may exhibit more innocuous conditions than ACS, including stable coronary artery disease or pulmonary causes such as pneumonia [3]. Prehospital triage is crucial for subsequent allocation and timely treatment to improve outcome but also to preserve resources [4].

In the prehospital emergency setting diagnostic means are limited to clinical assessment and electrocardiographic (ECG) evaluation in the absence of laboratory results. Therefore, emergency physicians have to rely on current complaints, ECG findings, and previous medical history to decide on prehospital treatment and prompt allocation to a suitable hospital. However, diagnostic uncertainty remains, as confirmation of ACS by laboratory and radiographic findings (i.e., coronary angiography) is lacking [5]. Scoring tools to define high risk patients, e.g., the HEART score, are not suitable in the German prehospital setting, since they also require laboratory results [6].

Although ACS is a common condition in prehospital emergency medicine, data on diagnostic specificity are limited and divergent, with a range between 46% and 80% accuracy [7, 8]. Furthermore, to our knowledge, data on kind and frequency of misdiagnoses are missing.

Therefore, the aims of the present study were to evaluate the diagnostic accuracy of ACS in a physician based prehospital emergency setting, to define common differential diagnoses, and to delineate possible discriminating features which may help to improve diagnostic accuracy.

## 2. Materials and Methods

### 2.1 Study Design

In accordance to the Declaration of Helsinki and §15 of the Medical Association Nordrheins’ professional code of conduct, we retrospectively reviewed all patients treated by a physician-staffed Emergency Medical Team (PEMT) at the prehospital emergency medicine department of Bonn between January 1st 2018 and December 31st 2018 (Ethics Committee of the University Hospital Bonn, Germany Approval No. 055/22) to evaluate the accuracy of ACS diagnoses.

### 2.2 Setting

In Germany, the EMS include PEMT that are dispatched to the scene in addition to ambulances depending on case severity. At the EMS of Bonn, approximately 320,000 residents are supplied by three PEMTs in addition to 17 ambulances.

The dispatch center allocates both PEMTs in addition to paramedic staffed ambulances towards the scene, if the emergency call is consistent with an ACS. In case a sole paramedic team suspects an ACS upon arrival on scene, a physician is to be requested additionally. Based on institutional standards, in every case of chest pain a 12 lead ECG has to be acquired at the scene and establishment of a venous catheter in addition to baseline monitoring (serial noninvasive blood pressure measurement, continuous pulsoxymetry, body temperature, blood glucose) is required. If this primary survey is consistent with ACS, the patient requires hospital admission to a cardiology department with an available acute coronary angiography suite. Administration of heparine and/or acetylsalicylic acid (ASA) were subject to medication history and the physician’s assessment.

Medical records of all patients that have been treated by a PEMT between January 1st 2018 and December 31st 2018 were retrospectively reviewed to identify all cases with prehospitally suspected ACS. All patients were diagnosed and treated by an emergency physician according to international guidelines and institutional standards and subsequently transferred to a hospital. Patients who refused or did not require hospital admission were excluded, as were patients necessitating cardiopulmonary resuscitation.

From medical records date, time, and location of emergency were extracted as well as medical information such as patient age and sex, medical history, presenting symptoms, vital signs, ECG findings, and administered drugs.

Discharge records from allocated hospitals were obtained to validate prehospital diagnosis. Further investigations to confirm or rule out ACS were subject to hospital protocols. Depending on symptoms upon hospital admission, laboratory results (i.e., high-sensitive troponin), and evaluation of both prehospital and in-hospital ECG findings, patients received coronary angiography, cardiac CT or neither.

### 2.3 Statistical Analysis 

Descriptive statistics are presented with numbers and percentages (%) for categorical variables and means with standard deviations (sd) for continuous variables. The exploratory statistical analysis follows a two-step approach: First, group differences (ACS vs. nACS) regarding patient characteristics and pre-clinical information are assessed via univariate statistical tests using non-parametric Wilcoxon Mann-Whitney tests for continuous variables and Fisher’s exact tests for categorical variables together with unadjusted odds-ratios (OR) and corresponding two-sided 95% confidence intervals (CI). Two-sided *p*-values below 0.05 are considered statistically significant and no adjustment is performed for multiple testing due to the exploratory nature of the analysis. Afterwards, a data-driven selection of informative predictors of confirmed ACS is performed via the lasso penalized regression technique [9]. A multivariable logistic regression model with confirmed ACS as outcome and all available prehospital information as potential predictors is estimated while tuning the penalization term (lambda) via 10-fold cross-validation. Missing values in the candidate predictors were imputed with the sample mean for this analysis. The lasso imposes shrinkage of effect estimates towards zero and therefore automatically leads to the selection of informative predictors in a data-driven manner. The resulting adjusted odds-ratios (adj. OR) from the model coefficients are reported for the selected variables.

## 3. Results

During the evaluation period of one year, 837 patients were treated by a PEMT with presumed ACS and admitted to a hospital for continuing diagnostic and subsequent therapy. 79 patients were excluded from analysis because in-hospital documentation was not accessible.

**Fig. 1.**
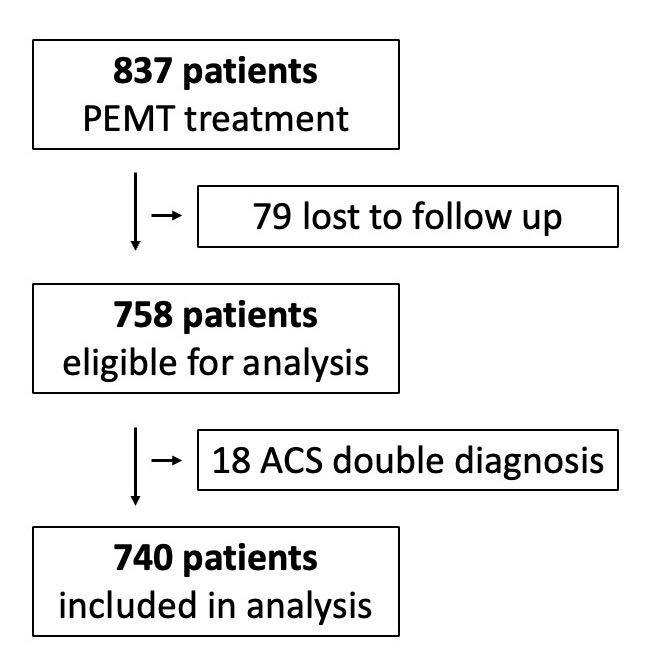
**Exclusion criteria for analysis**.

In 18 cases the final diagnosis was a composite of ACS in addition to other diagnoses. These patients were excluded to impede confounding factors, and therefore 740 patients remained for final analysis (Fig. 1). Included patients had a mean age of 69.2 years, ranging between 18 and 98 years. Most patients were treated singularly, while 40 patients were treated more than once (range 2–4 times).

Prehospitally suspected ACS was verified by cardiac troponin serum analysis or coronary angiographic evaluation during the clinical course in 283 out of 740 patients (38.2%, ACS group). However, 457 patients (61.8%) received a final diagnosis that was not ACS and thus constitute the non-ACS group (nACS group).

The ACS group consisted of significantly more males (68.2% vs. 49.7%; OR 2.17; *p *< 0.001) as compared to the nACS group. However, groups did not differ regarding age or number of antiaggregant drugs. A history of atrial fibrillation (AF) was found more often in the nACS group as compared to the ACS group (22.7% vs. 14.2%; OR 0.57; *p* = 0.005). Other pre-existing cardiac disorders, i.e., cardiomyopathy, myocarditis, valvular disease, or arrhythmia other than AF, were also more frequent in the nACS group (12.3% vs. 5.0%; OR 0.37; *p* = 0.001). Further details on baseline characteristics are presented in Table 1.

**Table 1.** **Baseline characteristics**.

		ACS group	nACS group	*p*-value	OR (95% CI)	Missings
		(n = 283)	(n = 457)			
Sex				*p <* 0.001	2.17 (1.58–3.0)	0
	Male	193 (68.2%)	227 (49.7%)			
	Female	90 (31.8%)	230 (50.3%)			
Age [years, mean, sd]		70.7 (± 15.9)	68.3 (± 15.9)	*p* = 0.16		0
Pre-existing cardiovascular conditions						
	Ischemic heart disease	121 (43.1%)	179 (39.4%)	*p* = 0.35	1.16 (0.85–1.59)	5
	Atrial fibrillation	40 (14.2%)	103 (22.7%)	*p = *0.005	0.57 (0.37–0.86)	5
	Arterial hypertension	195 (69.4%)	308 (67.8%)	*p* = 0.68	1.07 (0.77–1.5)	5
	Diabetes	60 (21.4%)	97 (21.4%)	*p* = 1	1.00 (0.68–1.46)	5
	Other conditions	14 (5.0%)	56 (12.3%)	*p =* 0.001	0.37 (0.19–0.7)	5
Number of anticoagulant agents [mean, sd]		0.7 (± 0.7)	0.7 (± 0.7)	*p* = 0.91		43

As shown in Table 2, occurrence of classic clinical angina pectoris (AP) was not different between ACS und nACS groups (83.5% vs. 79.0%; OR 1.35; *p* = 0.15). Also, presentation with atypical thoracic pain (OR 1.53; *p* = 0.17) or dyspnea (OR 1.03; *p* = 0.93) was similar between groups. However, ST segment elevations occurred significantly more often in the ACS group (38.5% vs. 14.8%; OR 3.59; *p *< 0.001) as did ST segment depressions (30.1% vs. 22.6%; OR 1.47; *p* = 0.05).

**Table 2.** **Prehospital patient characteristics**.

		ACS group	nACS group	*p*-value	OR (95% CI)	Missings
		(n = 283)	(n = 457)			
Symptoms on presentation						
	Angina pectoris	233 (83.5%)	357 (79.0%)	*p* = 0.15	1.35 (0.9–2.04)	9
	Atypical thoracic pain	23 (8.2%)	25 (5.5%)	*p* = 0.17	1.53 (0.81–2.88)	9
	Dyspnea	68 (24.4%)	108 (23.9%)	*p* = 0.93	1.03 (0.71–1.47)	9
	Syncope	6 (2.1%)	27 (6.1%)	*p =* 0.02	0.33 (0.11–0.84)	14
	Headache	3 (1.1%)	19 (4.3%)	*p =* 0.01	0.24 (0.04–0.82)	14
Findings on presentation						
	ST-elevation	92 (38.5%)	55 (14.8%)	*p <* 0.001	3.59 (2.4–5.4)	129
	ST-depression	72 (30.1%)	84 (22.6%)	*p =* 0.05	1.47 (1.0–2.16)	129
	T wave changes	22 (9.2%)	23 (6.2%)	*p* = 0.2	1.53 (0.79–2.95)	129
	GCS <15	3 (1.1%)	17 (4.0%)	*p =* 0.03	0.27 (0.05–0.94)	42
	Systolic blood pressure [mmHg, mean, sd]	147 (± 32)	149 (± 31)	*p* = 0.76		1
	Heart rate [mean, sd]	86 (± 21)	88 (± 24)	*p* = 0.23		3
Prehospital treatment						
	Heparin	221 (78.1%)	292 (63.9%)	*p <* 0.001	2.01 (1.42–2.88)	0
	ASA	205 (72.4%)	288 (63%)	*p =* 0.008	1.54 (1.1–2.16)	0
	Nitroglycerin	93 (32.9%)	134 (29.4%)	*p* = 0.33	1.18 (0.84–1.64)	1

Abbreviations: ASA, acetylsalicylic acid; GCS, Glasgow Coma Scale.

Occurrence of neurological symptoms including syncope was significantly more frequent in the nACS group (6.1% vs. 2.1%; OR 0.33; *p* = 0.02) as was presentation with headaches (4.3% vs. 1.1%; OR 0.24; *p* = 0.01). Similarly, an impaired neurological status indicated by a reduced Glasgow Coma Scale (GCS) score was more prevalent in the nACS group (OR 0.27; *p* = 0.03).

Analyzing data from in-hospital records including clinical symptoms and ECG alterations showed concordant results with pre-hospital findings. In the clinical setting ST segment elevation (38.5% vs. 4.8%; OR 13.21; *p *< 0.001) and ST segment depressions (24.7% vs. 7.4%; OR 4.29; *p *< 0.001) were more common in ACS group compared to nACS group. Elevated high-sensitive cardiac Troponin T was significantly more frequent in the ACS group (60.1% vs. 19.7%; OR 6.91; *p *< 0.001). Coronary angiography was performed in 91.9% of patients in the ACS group as compared to 21.4% in the nACS group (OR 40.83; *p *< 0.001).

Final diagnoses in the nACS group included a variety of diseases. A majority of patients were diagnosed with non-specific thoracic pain (145 patients, 31.7%). In 113 patients (24.7%) the ultimate diagnosis was cardiologic, mainly arrhythmias and decompensated heart failure. Hypertensive emergency was diagnosed in 73 patients (16%), abdominal causes in 50 patients (10.9%), and pulmonary causes in 36 patients (7.9%). Further details can be seen in Table 3.

**Table 3.** **Final diagnoses in nACS group**.

Final diagnoses in nACS group (n = 457)		
(multiple diagnoses per patient were possible)		
113 cardiologic		
	54	arrhythmia
	31	decompensated heart failure/cardiogenic shock
	9	valvular disease
	9	myocarditis/pericarditis/endocarditis
	13	various
50 abdominal		
	25	gastritis/gastroesophageal reflux disease/gastroenteritis
	9	pancreatitis/cholecystitis/urinary tract infection
	6	acute kidney injury
	10	various
37 pulmonary		
	15	respiratory tract infection
	9	restrictive/obstructive ventilation disorder
	6	pulmonary embolism
	9	various
10 neurologic		
38 infectious diseases		
262 various		
	145	unspecific pain
	76	hypertensive emergency
	16	syncope
	7	psychiatric/intoxication
	5	traumatic
	15	various

In order to identify potentially predictive prehospital parameters for accurate diagnosis of ACS, multivariable analysis with data-driven variable selection was performed using the lasso technique. Incorporating all prehospital available variables as potential candidates, the lasso selected ST segment elevation (adj. OR 2.70), male sex (adj. OR 1.71), T inversion (adj. OR 1.27), AP (adj. OR 1.15), ST segment depression (adj. OR 1.14), repetitive PEMS treatment (adj. OR 1.02), age (adj. OR 1.01 per year), implanted devices (adj. OR 0.94), a history of atrial fibrillations (adj. OR 0.76), headache (adj. OR 0.72) and a syncope (adj. OR 0.63) as informative predictors for ACS.

## 4. Discussion

Acute coronary syndrome is a common condition with relevant morbidity and mortality. During the reviewed period of one year, the EMS Bonn responded to a total of 9.259 PEMT calls. In 837 cases (9%), this was for suspected ACS, demonstrating its high occurrence. A Swiss study analyzed main complaints to demand PEMT services over a 10 year period, with chest pain being the reason in 5.9%–7.7% [2]. The incidence in this reported study is marginally higher, and one would assume that for an experienced emergency physician diagnosis of a common disease would be simple, but available data are scarce and incongruent.

Our study revealed that 283 out of 740 patients (38.2%) were correctly diagnosed. Prehospital diagnosis is based on presenting symptoms, clinical examination, patient’s previous history, and ECG alterations in the absence of laboratory diagnostics, possibly leading to a surprisingly high rate of falsely assumed ACS. Additionally, PEMTs rather suspect than miss an ACS. The observed proportion of correct ACS diagnoses is similar to an Austrian study, but significantly lower than a previously published retrospective analysis from our department evaluating overall correct prehospital diagnosis [7, 8]. Explaining these diverging results remains speculative. Analyses from Schewe *et al*. [8] were calculated based on voluntarily submitted discharge letters from the treating hospital, leading to a follow-up rate of just 25%. One might speculate that only relevant results (e.g., myocardial infarction) were reported back, while inconclusive in-hospital findings were not submitted, thus leading to a false-positive result of relevant diagnoses in the previous study. However, in the presented study follow up was obtained for 90% of all treated patients.

Interestingly, in this cohort angina pectoris is selected as an informative predictor using the multivariable lasso model (adj. OR 1.15) but no significant difference was found in the univariate analysis (*p* = 0.15) comparing the two groups. This only seems contradictory, but underlines the fact, that a sole symptom does not diagnose an ACS (univariate analysis), but should be considered seriously if combined with male sex, ST segment changes or T inversions (multivariable lasso model).

This is in line with the HEART score, that includes patient history, ECG changes, age, risk factors, and cardiac troponin measures, but not thoracic pain as sole symptom [6]. Also in concordance with the HEART score are ST-segment deviations on ECG, which were significantly more common in the ACS group. Although the HEART score is validated for in-hospital use to estimate the risk of major adverse cardiac events, a Dutch group confirmed its pre-hospital feasibility to stratify NSTEMI-ACS patients [6, 10].

Point of care diagnostics for cardiac troponin measurements have not been established in EMS across the board and are unavailable at the EMS Bonn. False negative test results need to be considered since troponin serum levels increase only hours after ACS onset. Additionally, the time to test result may take up to 20 minutes. Thus, point of care diagnostics may be helpful in a rural setting with long distances to a suitable hospital, but potentially delaying final treatment in metropolitan areas. Especially combined with the prehospital use of the HEART score, point of care troponin tests might be helpful, but are not recommended in current guidelines [11].

Serial measurement of high-sensitive troponin is mandatory in ACS diagnosis [5]. In the presented groups, ACS without elevated troponin as well as ruled out ACS with elevated troponin have been observed. Partly, this has methodical reasons, as only the first measured troponin was included into analysis. For accurate diagnosis, a second troponin measurement is necessary to evaluate the trend. This also explains troponin negative ACS in our analysis. Secondly, troponin was stratified binary, because analysis was laid out for a prehospital setting. Within the hospital context, mildly elevated troponin levels without further increase are commonly seen in renal insufficiency, arrhythmias or decompensated heart failure, not resulting in an ACS diagnosis. Depending on ECG findings and clinical presentation, coronary angiography was performed for a definite diagnosis and possible intervention.

Studies on atypical ACS presentation in women lead to contradictory results. While some report atypical pain radiation, others show no significant sex differences [12, 13, 14]. In our study, women had significantly less confirmed ACS as compared to men (*p *< 0.001; OR 2.17), but there was no sex difference in the nACS group.

Nonspecific chest wall pain, gastroesophageal reflux, pneumonia, heart failure, or pulmonary embolism are well known differential diagnoses and have been found in the nACS group [15]. With the exception of pulmonary embolism, loading doses of heparin and acetylsalicylic acid (ASA) recommended for ACS would not be indicated [16]. In the nACS group, 63.9% of patients received heparin, and 63% ASA, potentially inducing bleeding complications in these patients.

In the nACS group, two patients were ultimately diagnosed with acute aortic dissection of which one was treated with heparin and ASA, which is contraindicated and potentially results in desastrous hemorrhage [17]. Two cases were intracranial hemorrhages, being treated with contraindicated heparin and ASA. Neurological diseases masked as ACS have been well described and are subsumed as neurogenic stunned myocardium [18]. Although difficult to distinguish, PEMTs have to consider these differential diagnoses in order to prevent incorrect, possibly harmful, treatment.

In the presented cohort, one suspected case of ACS proved to be systemic lupus erythematosus. This is a rare differential diagnosis, but might be explained due to coronary vasculitis and has been described before [19]. One case of pheochromocytoma was observed, in which the catecholamine surge may explain symptoms mimicking an ACS [20]. Other rare cases were upside-down stomach and fibromyalgia, demonstrating the range of differential diagnoses for ACS.

Patients with presumed but not confirmed ACS were treated and allocated incorrectly. This may lead to capacity overload of chest pain units and coronary angiography suites. Additionally, nACS patients may require an interhospital transfer for adequate treatment, also delaying therapies and tying up capacities. Although hospital admission was necessary for the majority of evaluated patients, allocation to a hospital without chest pain unit, monitoring capacity, and coronary angiography suite would have sufficed for most of the misdiagnosed patients. For ACS patients, a correct diagnosis as early as possible is decisive for adequate and time sensitive therapy. Scores estimating severity and mortality in chest pain patients such as TIMI (Thrombolysis In Myocardial Infarction) and GRACE (Mini-Global Registry of Acute Coronary Events) are neither validated in the prehospital setting, nor applicable, since they rely on laboratory results [11].

Although in the prehospital setting a definitive ACS diagnosis might be impossible, PEMTs have to estimate the probability. The misdiagnosis rate of 62% in this evaluated cohort is too high, supporting the importance of known risk factors (i.e., male sex) and diagnostic findings (i.e., ST segment changes). The majority of patients in the nACS group (79%) presented with AP. Therefore, chest pain needs to be interpreted in the context of further findings and history, and ACS presenting with chest pain only should be questioned. Even more important, signs inconsistent with ACS, in our series neurological symptoms, need to be considered as “red flags” for differential diagnoses.

## 5. Limitations

Results from the present exploratory analysis must be interpreted with caution due to the retrospective and monocentric design of this study. While for 79 patients in-hospital documentation was unavailable, prehospital ECG documentation was incomplete in 129 cases (17.4%). Although emergency physicians at the EMS Bonn all went through a thorough training including ECG interpretation, they are mainly not cardiologists.

Note the fact that a potential predictor variable does not yield a significant difference between the two groups (e.g., AP) but is still selected via the lasso as an informative predictor is only seemingly contradictive. While the significance testing is based on univariate associations (testing the null hypothesis of no association), the lasso model is based on a multivariable approach aiming to find a suitable prediction model combining the effect of various predictors. The lasso approach must be seen as fully exploratory, the fact that a variable is selected is no confirmation of the underlying effect. On the other hand, the fact that a variable does not yield a significant group difference only shows that there is not enough evidence to reject the null hypothesis, but does not confirm that the effect does not exist.

## 6. Conclusions

This retrospective single center analysis reveals in the prehospital setting a high proportion (61.8%) of incorrectly suspected ACS patients with a wide range of differential diagnoses. From a multivariable approach using the lasso technique, ST-segment elevations (adj. OR 2.70) combined with male sex (adj. OR 1.71), T wave changes (adj. OR 1.27) and AP (adj. OR 1.15) seem indicative of ACS. Most interesting, presentation with neurological symptoms should be considered as a “red flag” for the emergency physician. Thus, an attentive examination and history are highly important for the emergency physician to supply adequate and timely therapy, avoid inappropriate prehospital therapy and reduce occupation of specialized ressources necessary for an actual ACS.

## Data Availability

The datasets used and analyzed during the current study are available from the corresponding author on reasonable request.

## Abbreviations

ACS, acute coronary syndrome; AF, atrial fibrillation; AP, angina pectoris; ASA, acetylsalicylic acid; ECG, electrocardiogram; EMS, emergency medical service; GCS, Glasgow Coma Scale; PEMT, physician-staffed emergency medical team.
